# Supplementary material for: USP15-dependent lysosomal pathway controls p53-R175H turnover in ovarian cancer cells
Source: Nat Commun. 2018 Mar 28;9:1270. doi: 10.1038/s41467-018-03599-w (PMC5871815; doi:10.1038/s41467-018-03599-w)
Supplement: Supplementary file 1 — Supplementary Information(PDF 3917 kb) [file 41467_2018_3599_MOESM1_ESM.pdf]

Supplementary Information

USP15-dependent lysosomal pathway controls p53-R175H turnover in ovarian cancer cells

Padmanabhan et.al.

Supplementary Figures

A

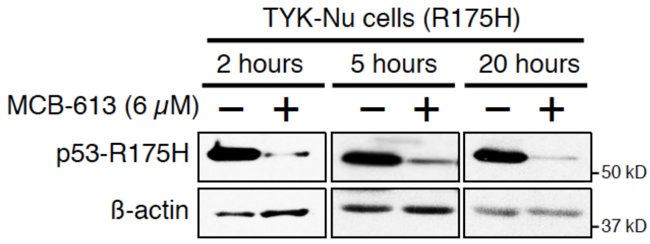

B

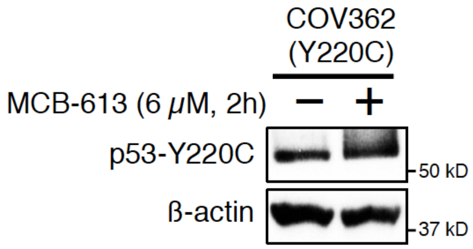

C

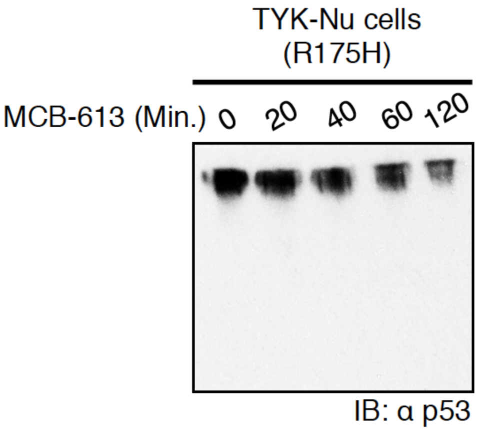

Supplementary Figure 1. MCB-613 causes a rapid and selective decrease in the level of p53-R175H.
(A) MCB-613 (6 μM) causes sustained decrease in p53-R175H levels in TYK-Nu cells.
(B) MCB-613 (6 μM, 2h) does not alter the levels of p53-Y220C in COV362 cells.
(C) Native gel-western blot analysis showing the levels of p53-R175H is reduced upon MCB-613 treatment in a time-dependent manner.

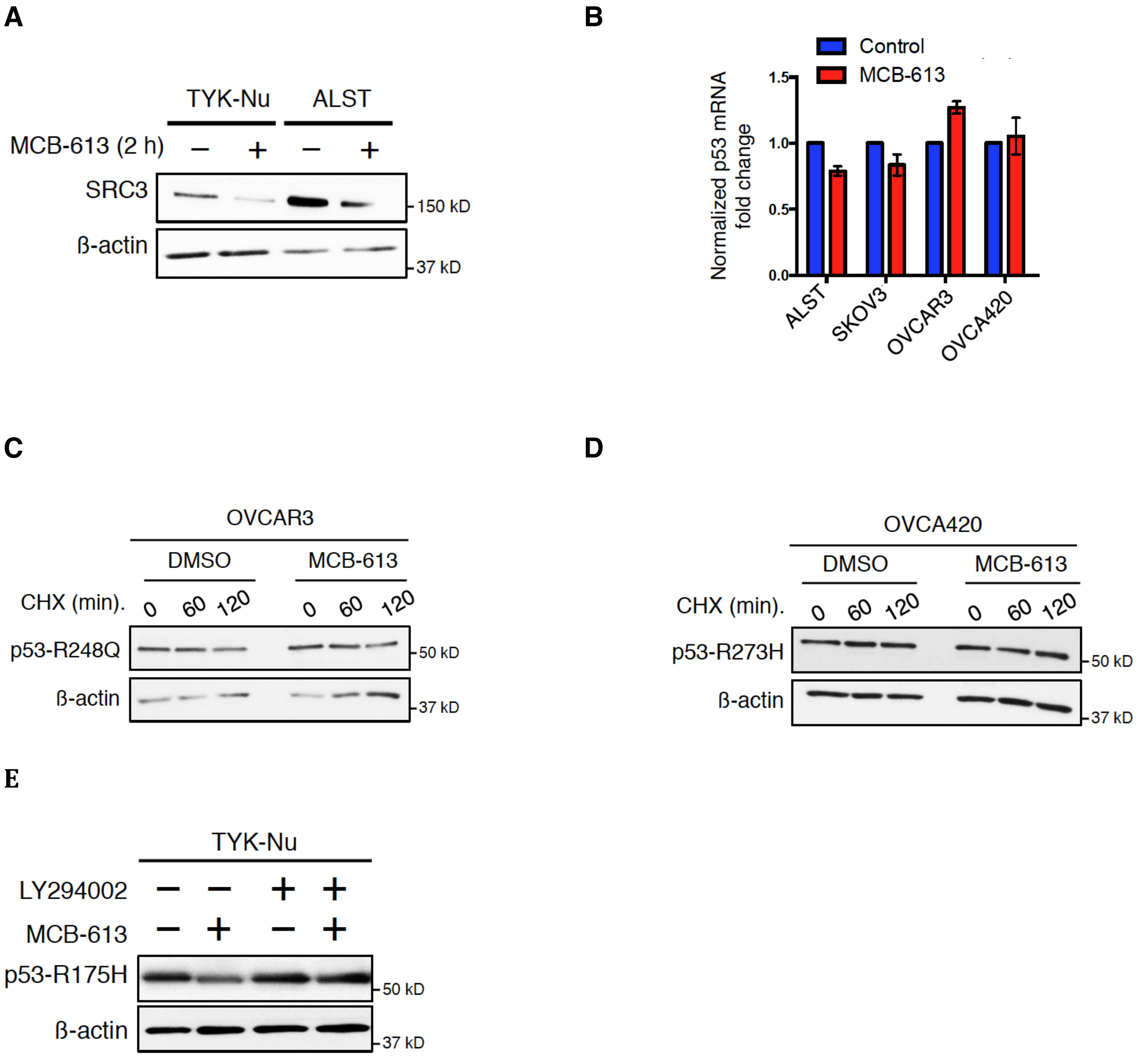

Supplementary Figure 2. MCB-613 has no effect on the half-life of p53-R248Q and p53-R273H.

(A) MCB-613 treatment (6 μM, 2h) causes a decrease in SRC3 levels in both TYK-Nu and ALST cells.

(B) Effect of MCB-613 (6 μM, 2h) on p53 mRNA levels in ALST, SKOV3, OVCAR3 and OVCA420 cells. The data for ALST cells is the same as that in Figure 2A and has been included here for comparison. Values are mean $\pm$ s.e.m (N=3).

(C and **D)** Cycloheximide chase analysis show that MCB-613 (6 μM) has no effect on the turnover of (**C**) p53-R248Q mutant protein in OVCAR3 cells and (**D**) p53-R273H mutant protein in OVC420 cells.

(E) The effect of MCB-613 on p53-R175H is reversed upon co-treatment with the autophagy inhibitor LY294002.

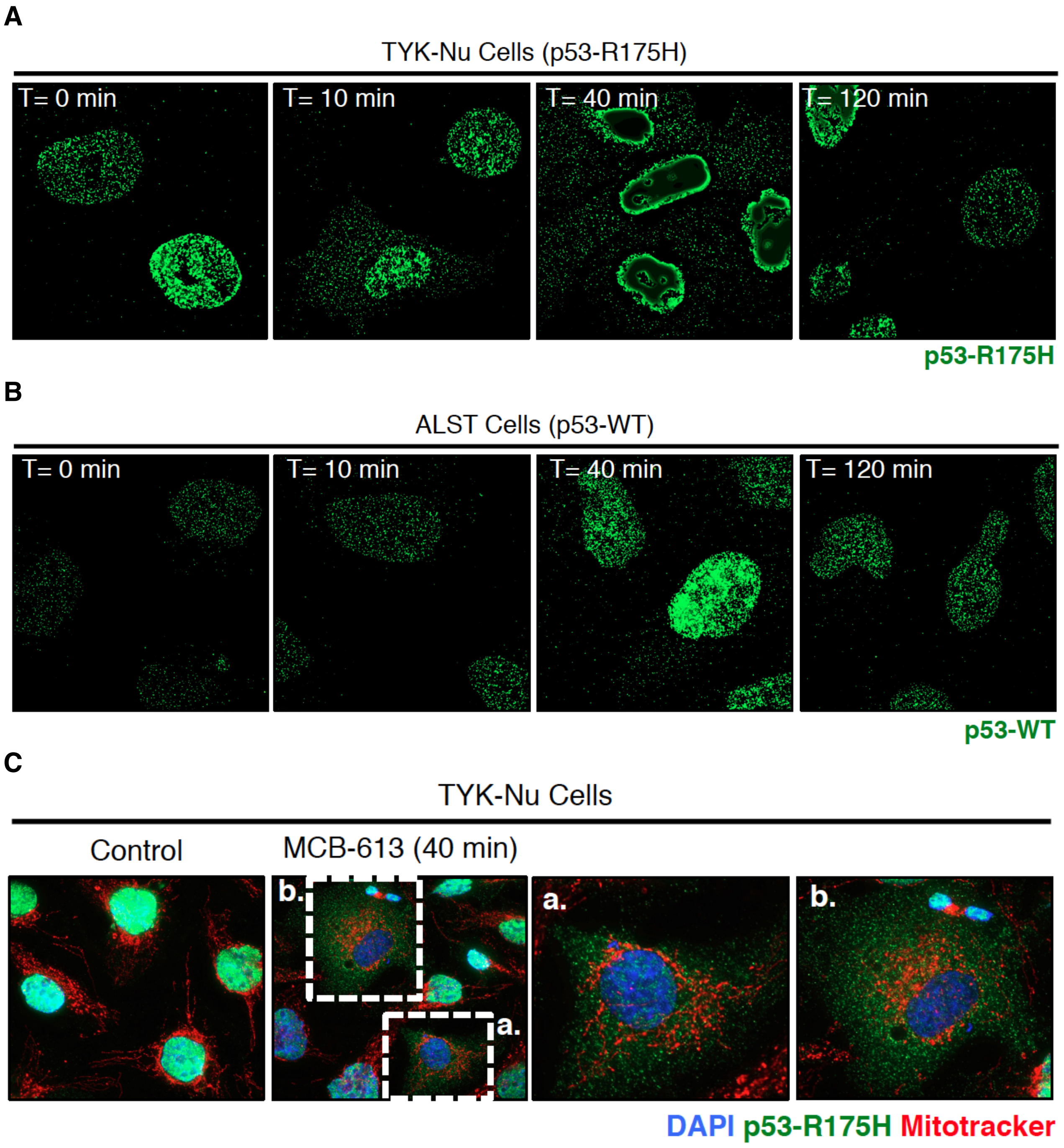

Supplementary Figure 3. p53-R175H does not co-localize with mitochondria upon MCB-613 treatment.

(A) p53-R175H (green) protein in TYK-Nu cells localizes in the cytoplasm upon MCB -613 treatment. This figure shows the p53 (green) channel from figure 3A.

(B) Localization of p53-WT (green) protein in ALST is not altered upon MCB-613 treatment. This figure shows the p53 (green) channel from figure 3B.

(C) Co-immunofluorescence studies using TYK-Nu cells show absence of co-localization of p53-R175H (green) and mitochondria (mitotracker; red) upon MCB-613 treatment (6 μM, 40 min).

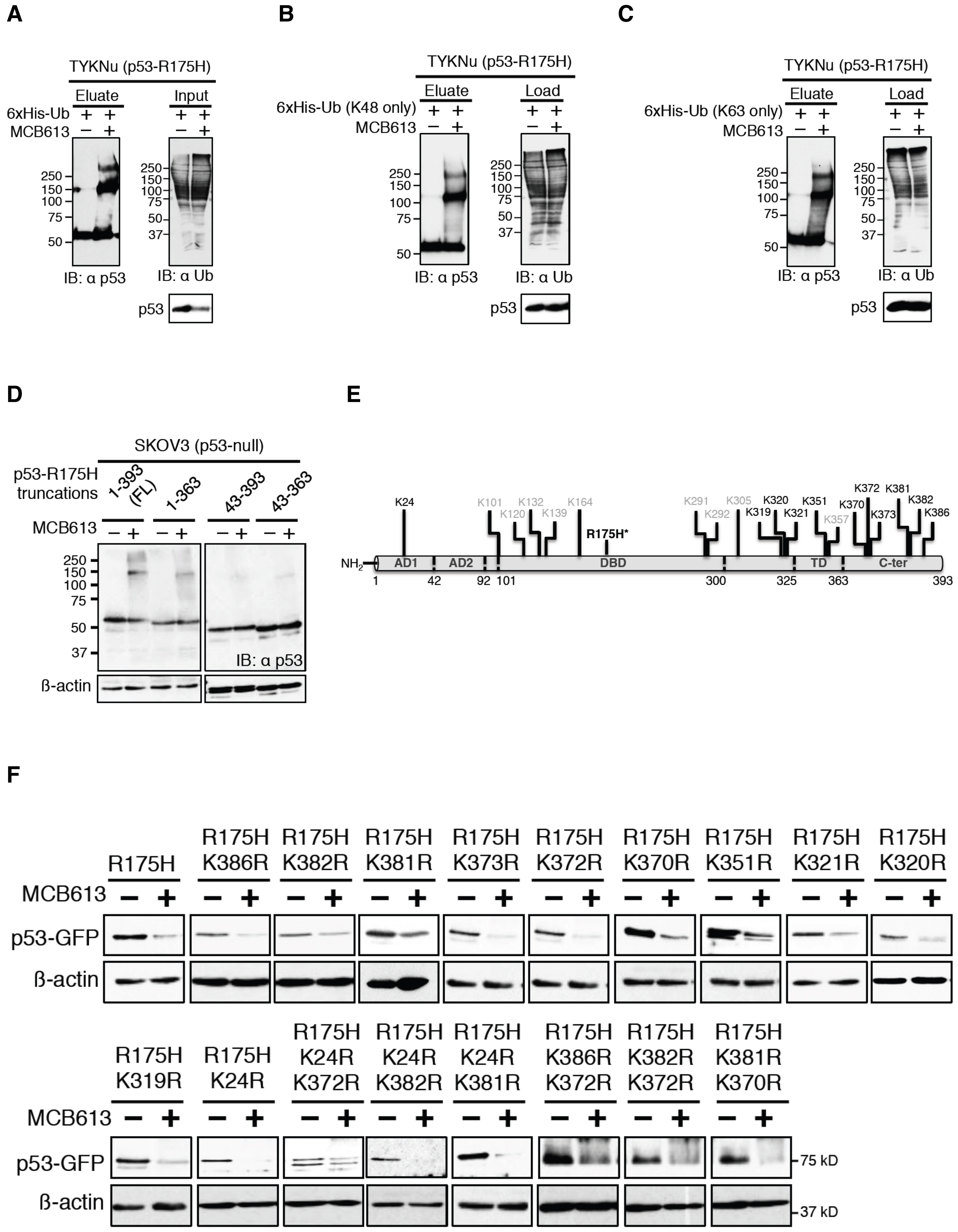
A
TYKNu (p53-R175H)
Eluate
Input
6xHis-Ub
MCB613
IB: α p53
IB: α Ub
p53
B
TYKNu (p53-R175H)
Eluate
Load
6xHis-Ub (K48 only)
MCB613
IB: α p53
IB: α Ub
p53
C
TYKNu (p53-R175H)
Eluate
Load
6xHis-Ub (K63 only)
MCB613
IB: α p53
IB: α Ub
p53
D
SKOV3 (p53-null)
p53-R175H truncations
1-393 (FL)
1-363
43-393
43-363
MCB613
IB: α p53
ß-actin
E
NH2
AD1
AD2
DBD
TD
C-ter
R175H*
K24 K101 K120 K132 K139 K164 K291 K292 K305 K319 K320 K321 K351 K357 K370 K372 K373 K381 K382 K386
1 42 92 101 300 325 363 393
F
R175H
R175H K386R
R175H K382R
R175H K381R
R175H K373R
R175H K372R
R175H K370R
R175H K351R
R175H K321R
R175H K320R
MCB613
p53-GFP
ß-actin
R175H K319R
R175H K24R
R175H K24R K372R
R175H K24R K382R
R175H K24R K381R
R175H K386R K372R
R175H K382R K372R
R175H K381R K370R
MCB613
p53-GFP
ß-actin
75 kD
37 kD

G

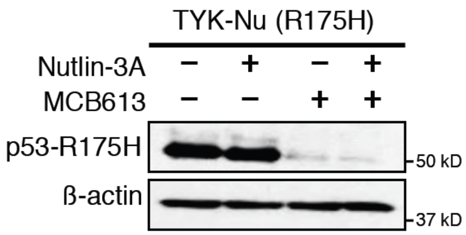

H

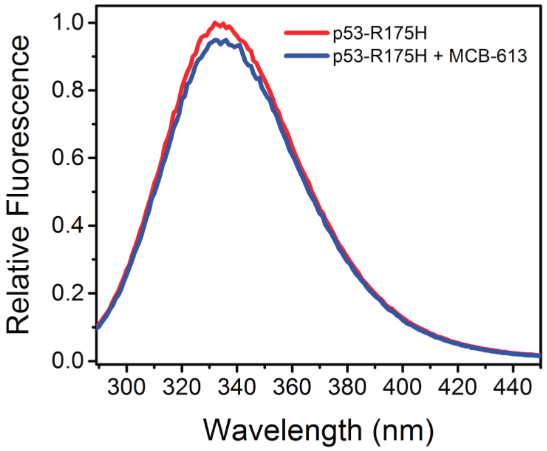

I

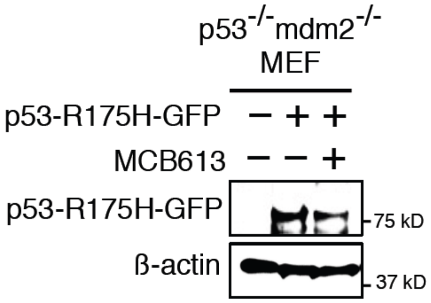

J

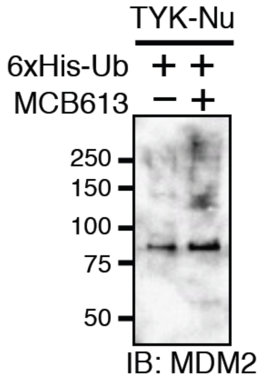

Supplementary Figure 4. Effect of MCB-613 on p53-175H is ubiquitination-dependent.
(A) Anti-p53 immunoblot on cellular proteins containing His-ubiquitin (pull down by Ni NTA chromatography) from TYK-Nu cells expressing ectopic 6xHis-Ub reveals increased ubiquitination of p53-R175H protein upon MCB-613 treatment.
(B) K-48 ubiquitin chains on p53-R175H protein is enhanced upon MCB-613 treatment in TYK-Nu cells.
(C) K-63 ubiquitin chains on p53-R175H protein is enhanced upon MCB-613 treatment in TYK-Nu cells.
(D) SKOV3 cells expressing carboxy and amino terminal deletion mutants of p53 R175H-HA (a kind gift from Dr. Wenwei Hu, Rutgers Univ., New Brunswick, NJ, USA) show that deletion of the amino terminal 42 amino acids or the carboxy terminal 30 amino acids from p53-R175H-HA mutant protein reverses the sensitivity of the R175H mutant to MCB-613.
(E) Cartoon showing lysine residues on p53 that can serve as potential ubiquitination sites. Sites that were mutated to arginine to test their role in MCB-613 induced turnover of p53-R175H is shown in black. Other potential ubiquitination sites are shown in grey. AD – Activation domain; DBD – DNA binding domain; TD – transactivation domain; C-ter – C-terminal domain. Amino acid positions are shown below.
(F) Effect of MCB-613 (6 μM, 2h) on ectopically expressed GFP-tagged p53-R175H protein containing additional lysine to arginine (K→R) substitution mutations (as indicated) in SKOV3 cells (p53-null).
(G) Effect of Nutlin-3A (10 μM, 2h) on p53-R175H levels in TYKNu cells either by itself or in combination with MCB-613 (6 μM, 2h).

(**H**) MCB-613 does not bind to p53-R175H protein. Fluorescence binding assay was performed using recombinant purified p53-R175H protein and MCB-613 (6 μM).
(**I**) Effect of MCB-613 on p53-R175H in p53-/-: mdm2-/- MEFs (a kind gift from Dr. Gigi Lozano, MD Anderson Cancer Center, Houston, TX, USA).
(**J**) MCB-613 causes increased ubiquitination of MDM2 in TYK-Nu cells. Anti-MDM2 immunoblot on cellular proteins containing His-ubiquitin (pull down by Ni-NTA chromatography) from TYK-Nu cells expressing ectopic 6xHis-Ub. This figure was generated from the same samples used in Supplementary Figure 4A.

A

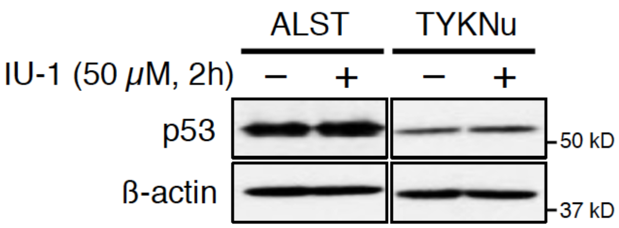

Supplementary Figure 5. USP14 inhibition has no effect of p53-WT or p53-R175H levels. **(A)** The USP14 specific inhibitor IU-1 (50 μM, 2h) had no effect on p53-WT in ALST cells and p53-R175H in TYK-Nu cells.

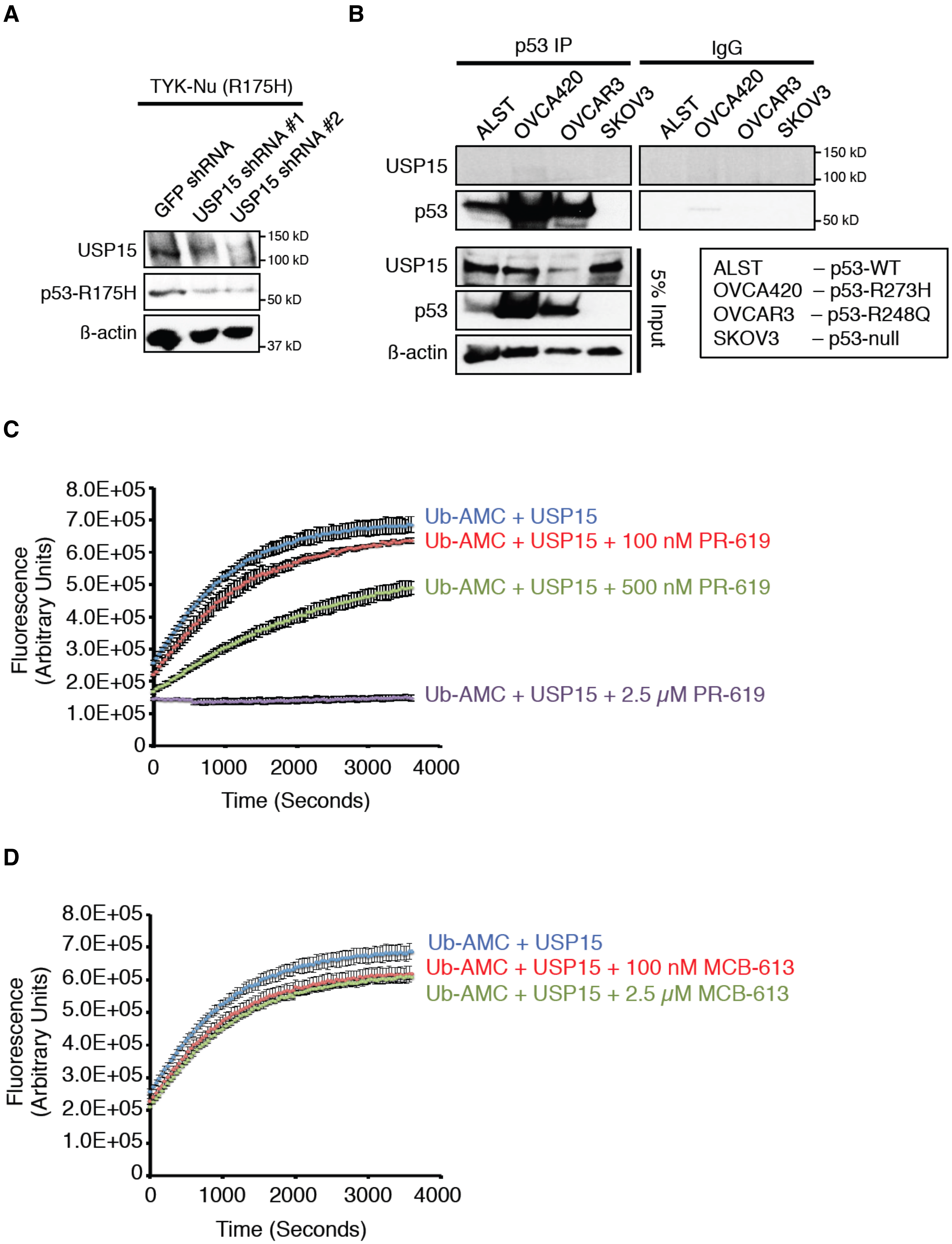
A
TYK-Nu (R175H)
GFP shRNA
USP15 shRNA #1
USP15 shRNA #2
USP15
p53-R175H
ß-actin
150 kD
100 kD
50 kD
37 kD
B
p53 IP
IgG
ALST
OVCA420
OVCAR3
SKOV3
USP15
p53
ß-actin
150 kD
100 kD
50 kD
5% Input
ALST – p53-WT
OVCA420 – p53-R273H
OVCAR3 – p53-R248Q
SKOV3 – p53-null
C
Fluorescence (Arbitrary Units)
Time (Seconds)
0
1.0E+05
2.0E+05
3.0E+05
4.0E+05
5.0E+05
6.0E+05
7.0E+05
8.0E+05
0
1000
2000
3000
4000
Ub-AMC + USP15
Ub-AMC + USP15 + 100 nM PR-619
Ub-AMC + USP15 + 500 nM PR-619
Ub-AMC + USP15 + 2.5 μM PR-619
D
Fluorescence (Arbitrary Units)
Time (Seconds)
0
1.0E+05
2.0E+05
3.0E+05
4.0E+05
5.0E+05
6.0E+05
7.0E+05
8.0E+05
0
1000
2000
3000
4000
Ub-AMC + USP15
Ub-AMC + USP15 + 100 nM MCB-613
Ub-AMC + USP15 + 2.5 μM MCB-613

E

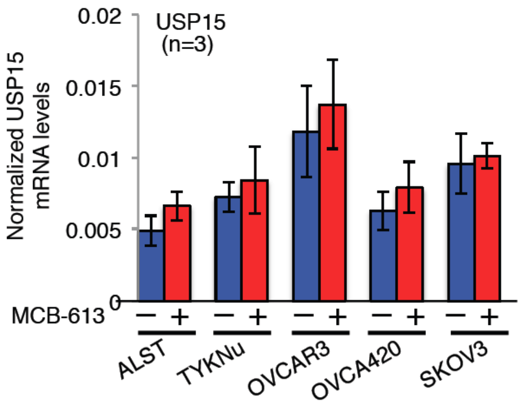

F

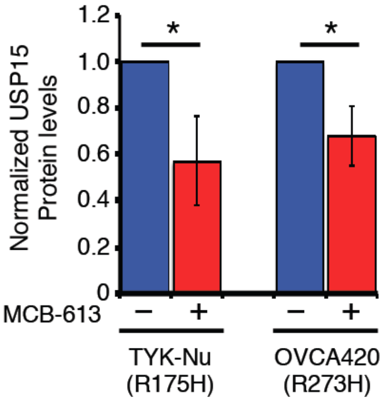

Supplementary Figure 6. Role of USP15 and USP7 in regulating the turnover of mutp53 proteins in ovarian cancer cells.

(A) USP15 knockdown using two different shRNA constructs results in decreased levels of p53-R175H protein in TYK-Nu cells.

(B) USP15 does not co-IP with p53-WT (in ALST cells), p53-R273H (in OVCA420 cells) and p53-R248Q (in OVCAR3 cells). p53-null SKOV3 cells was used in in as a control for non-specific binding of the p53 antibody (FL-393).

(C) DUB activity assay using Ub-AMC substrate shows PR-619 acts as a USP15 inhibitor. Values are mean $\pm$ s.e.m (N=3).

(D) DUB activity assay using Ub-AMC substrate shows MCB-613 does not inhibit USP15 catalytic activity. Values are mean $\pm$ s.em (N=3).

(E) Quantitative RT-PCR analyses shows that MCB-613 (6 μM, 2h) does not alter USP15 mRNA levels in the ovarian cancer cells carrying different p53 mutation status. USP15 mRNA levels were normalized using rpl19 mRNA levels in the corresponding samples. Values are mean $\pm$ s.d (N=3).

(F) Quantification of western blot analysis showing MCB-613 induces decrease in USP15 levels in both TYK-Nu and OVCA420 cells. The western blots were analyzed using imgae J and the USP15 expression was normalized to the corresponding ß-actin levels. Values are mean $\pm$ s.d (N=3; p-value <0.05).

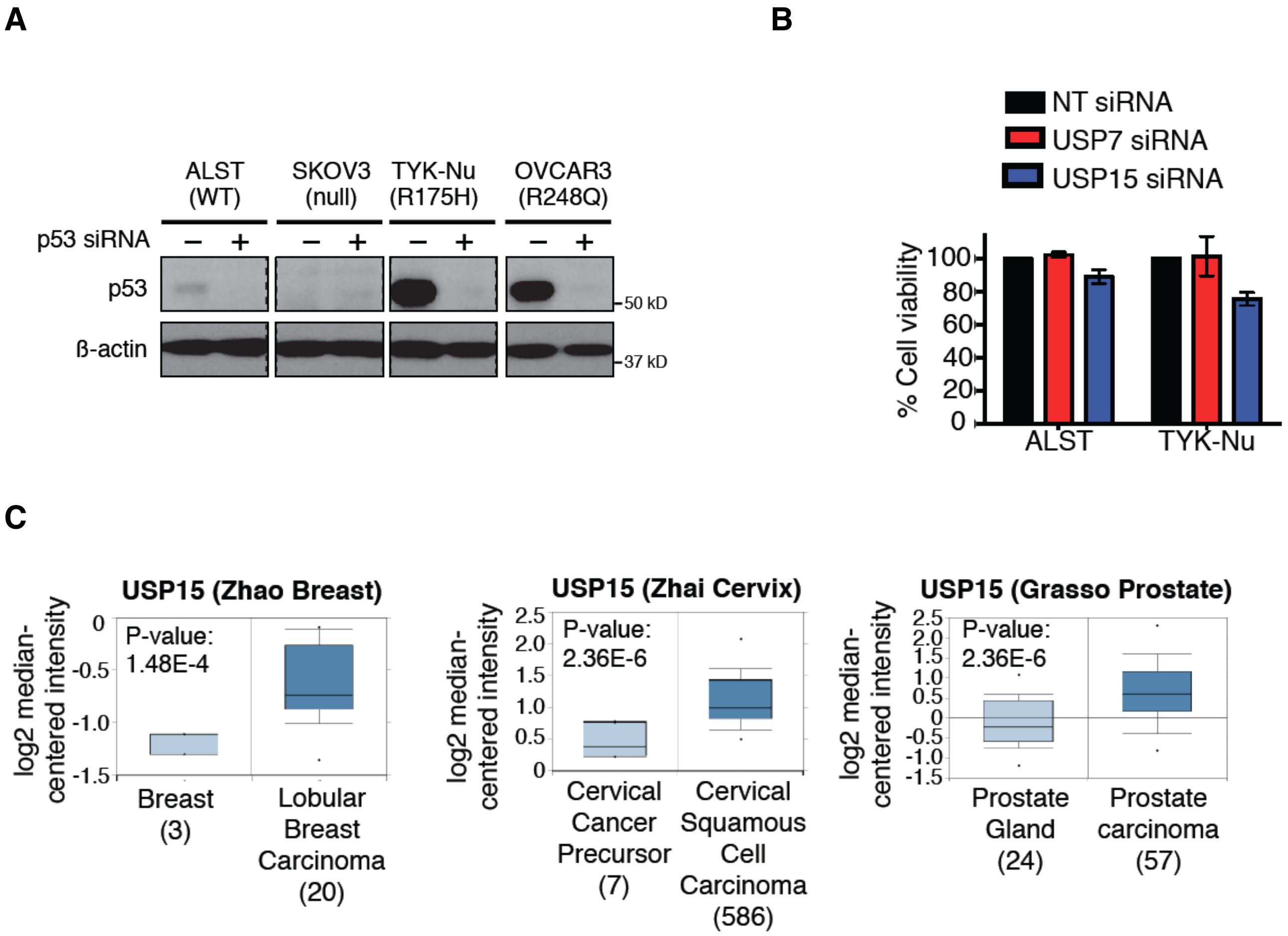

Supplementary Figure 7. USP15 depletion causes cancer cell death in ovarian cancer cells expressing p53-R175H.

(A) Western blot showing depletion of mutant p53 protein in cells treated with p53 siRNA.

(B) USP7 siRNA (48h) had no effect on cell viability in ALST and TYK-Nu cells. USP15 knockdown (48h) resulted in decreased viability of TYK-Nu cells. Values are mean $\pm$ s.e.m (N=3).

(C) Data retrieved from Oncomine showing elevated *USP15* mRNA levels in multiple cancers from previously published datasets[14, 23-26].

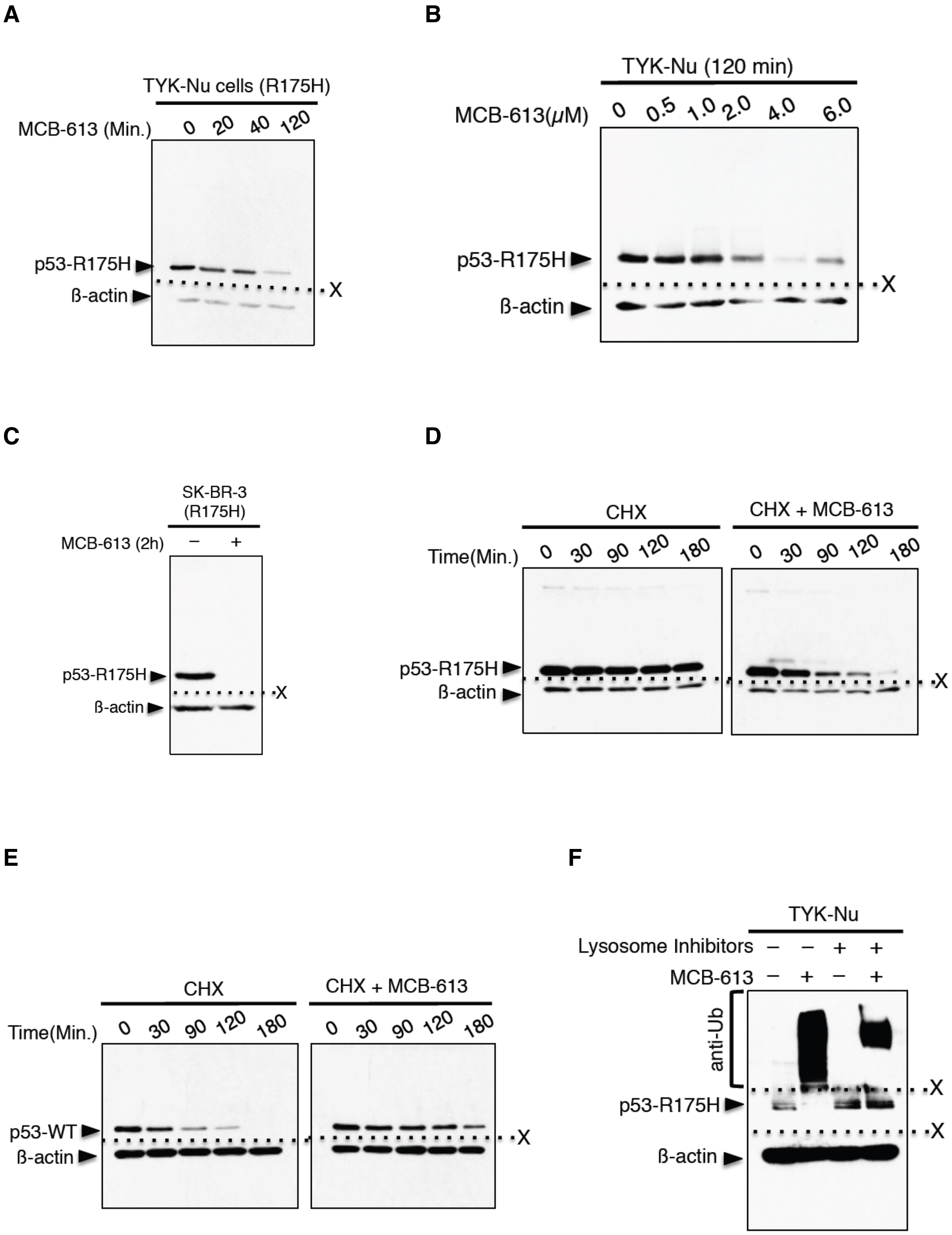
A
TYK-Nu cells (R175H)
MCB-613 (Min.)
0
20
40
120
p53-R175H
ß-actin
X
B
TYK-Nu (120 min)
MCB-613(μM)
0
0.5
1.0
2.0
4.0
6.0
p53-R175H
ß-actin
X
C
SK-BR-3 (R175H)
MCB-613 (2h)
−
+
p53-R175H
ß-actin
X
D
CHX
CHX + MCB-613
Time(Min.)
0
30
90
120
180
0
30
90
120
180
p53-R175H
ß-actin
X
E
CHX
CHX + MCB-613
Time(Min.)
0
30
90
120
180
0
30
90
120
180
p53-WT
ß-actin
X
F
TYK-Nu
Lysosome Inhibitors
−
−
+
+
MCB-613
−
+
−
+
anti-Ub
p53-R175H
ß-actin
X
X

G

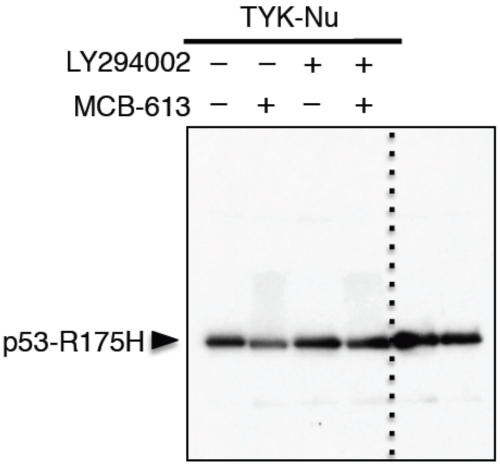

H

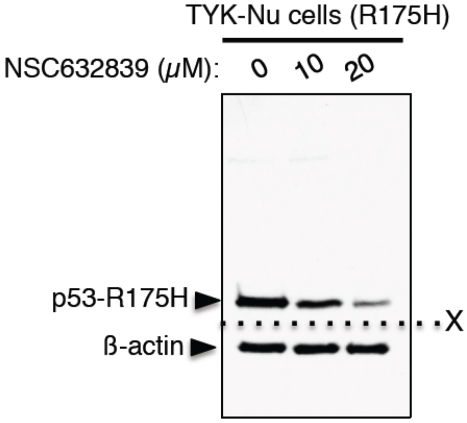

I

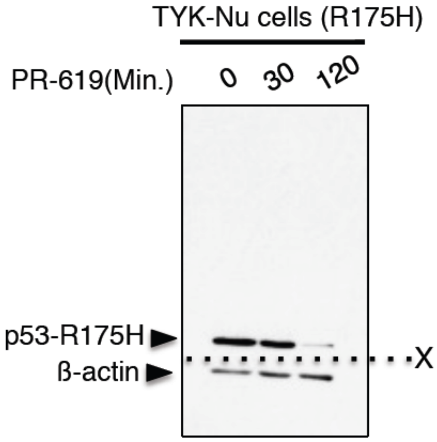

J

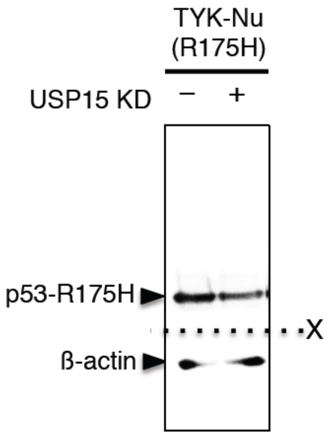

Supplementary Figure 8. Un-cropped scans of the key Western blots images in the paper.

(A) Un-cropped Fig. 1A. **(B)** Un-cropped Fig. 1B. **(C)** Un-cropped Fig. 1G. **(D)** Un-cropped Fig. 2G. **(E)** Un-cropped Fig. 2H. **(F)** Un-cropped Fig. 2J **(G)** Un-cropped Fig. S2E. **(H)** Un-cropped Fig. 5D. **(I)** Un-cropped Fig. 5F. **(J)** Un-cropped Fig. 6D. The dotted lines with 'x' at the end indicates the position at which the transferred PVDF membrane was cut to probe against different antibodies (as indicated). In Fig.S8G, the dotted line is used to indicate where the western blot was cropped. The PVDF membrane in Fig.S8G was not cut prior to performing Western blot.

Supplementary Tables :

Gene	Primer
p53-R248Q mutagenesis	Fwd: 5' CATGGGCGGCATGAACCAAAGGCCCATCCTCACCATC 3'<br>Rev: 5' GATGGTGAGGATGGGCCTTTGGTTCATGCCGCCCATG 3'
p53-K386R mutagenesis	Fwd: 5' CATAAAAAACTCATGTTCAGGACAGAAGGGCCTGACTC 3'<br>Rev: 5' GAGTCAGGCCCTTCTGTCCTGAACATGAGTTTTTTATG 3'
p53-K382R mutagenesis	Fwd: 5' CTCCCGCCATAAAAGACTCATGTTCAAGAC 3'<br>Rev: 5' GTCTTGAACATGAGTCTTTTATGGCGGGAG 3'
p53-K381R mutagenesis	Fwd: 5' CTACCTCCCGCCATAGAAAACTCATGTTCAAG 3'<br>Rev: 5' CTTGAACATGAGTTTTCTATGGCGGGAGGTAG 3'
p53-K373R mutagenesis	Fwd: 5' CCTGAAGTCCAAAAGGGGTCAGTCTACC 3'<br>Rev: 5' GGTAGACTGACCCCTTTTGGACTTCAGG 3'
p53-K372R mutagenesis	Fwd: 5' CCACCTGAAGTCCAGAAAGGGTCAGTCTAC 3'<br>Rev: 5' GTAGACTGACCCTTTCTGGACTTCAGGTGG 3'
p53-K370R mutagenesis	Fwd: 5' CCAGCCACCTGAGGTCCAAAAAGGG 3'<br>Rev: 5' CCCTTTTTGGACCTCAGGTGGCTGG 3'
p53-K351R mutagenesis	Fwd: 5' GAATGAGGCCTTGGAACTCCGAGATGCCCAGGCTGGGAAG 3'<br>Rev: 5' CTTCCCAGCCTGGGCATCTCGGAGTTCCAAGGCCTCATTC 3'
p53-K321R mutagenesis	Fwd: 5' CAGCCAAAGAAGAGACCACTGGATGGAG 3'<br>Rev: 5' CTCCATCCAGTGGTCTCTTCTTTGGCTG 3'
p53-K320R mutagenesis	Fwd: 5' CCCCAGCCAAAGAGGAAACCACTGGATG 3'<br>Rev: 5' CATCCAGTGGTTTCCTCTTTGGCTGGGG 3'
p53-K319R mutagenesis	Fwd: 5' CTCCCCAGCCAAGGAAGAAACCACTG 3'<br>Rev: 5' CAGTGGTTTCTTCCTTGGCTGGGGAG 3'
p53-K24R mutagenesis	Fwd: 5' CATTTTCAGACCTATGGAGGCTACTTCCTGAAAACAAC 3'<br>Rev: 5' GTTGTTTTCAGGAAGTAGCCTCCATAGGTCTGAAAATG 3'

Supplementary Table 1. List of primers used for site-directed mutagenesis.

Target protein	Vendor	Species	Cat. Number	Dilution
p53 (FL-393)	Santa Cruz	Rabbit	sc-6243	WB: 1:1000<br>IF : 1:250<br>IP : 1:50
p53 (DO-1)	Santa Cruz	Mouse	Sc-126	WB: 1:1000<br>IF : 1:250
SRC1	BD Transduction Laboratories	Mouse	612378	WB: 1:1000
SRC2	Bethyl Laboratories	Rabbit	A300-346A	WB: 1:1000
SRC3	Cell Signaling	Rabbit	2126S	WB: 1:1000
Ubiquitin	Cell Signaling	Rabbit	3933S	WB: 1:1000
Ubiquitin (P4D1)	Santa Cruz	Mouse	sc8017	WB: 1:1000
MDM2 (Ab-1)	Calbiochem	Mouse	OP46	WB: 1:1000
USP7	Bethyl Laboratories	Rabbit	A300-033A	WB: 1:1000
USP15 (B-5)	Santa Cruz	Mouse	sc-515688	WB: 1:1000
ß-actin (AC-74)	Sigma-Aldrich	Mouse	A-2228	WB: 1:5000
Calnexin	Abcam	Rabbit	ab75801	IF : 1:300

Supplementary Table 2. Details of list of antibodies used in this study. WB – Western blot ; IF – Immunofluorescence ; IP – Immunoprecipitation.

Target gene	Primer Sequence
p53	Fwd: 5' AAGTCCTTTGCCCTGAACTG 3'<br>Rev: 5' GGCTTGGAAGGCTCTAGG 3'
MDM2	Fwd: 5' TGTTGTGAAAGAAGCAGTAGCA 3'<br>Rev: 5' CCTGATCCAACCAATCACCT 3'
RPL19	Fwd: 5' GGTGACCTGGATGAGAAGGA 3'<br>Rev: 5' TTCAGCTTGTGGATGTGCTC 3'
USP15	Fwd: 5' CGACGCTGCTCAAAACCTC 3'<br>Rev: 5' TCCCATCTGGTATTTGTCCCAA 3'

Supplementary Table 3. List of primers used for qRT-PCR described in this study.

Sample name	Read count	Paired, mapped pairs %	Paired, broken pairs %	Paired, not mapped %
MCF-7 untreated-1	66656814	95.6	2.14	2.26
MCF-7 untreated-2	80456708	85.07	4.17	10.75
NSC632839 treated-1	42890788	81.35	14.68	3.96
NSC632839 treated-2	28255192	78.69	15.68	5.63
MCB-613 treated-1	32351076	74.75	17.54	7.71
MCB-613 treated-2	31792966	47.67	29.72	22.61

Supplementary Table 4. Read count statistics for RNAseq data.

Sample name	Mapped to genes %	Mapped to intergenic %
MCF-7 untreated-1	96.26	3.74
MCF-7 untreated-2	96.29	3.71
NSC632839 treated-1	97.54	2.46
NSC632839 treated-2	97.48	2.52
MCB-613 treated-1	97.39	2.61
MCB-613 treated-2	96.53	3.47

Supplementary Table 5. Fragment counting statistics for RNAseq data.
